# Mitochondrial Haplogroup H1 in North Africa: An Early Holocene Arrival from Iberia

**DOI:** 10.1371/journal.pone.0013378

**Published:** 2010-10-21

**Authors:** Claudio Ottoni, Giuseppina Primativo, Baharak Hooshiar Kashani, Alessandro Achilli, Cristina Martínez-Labarga, Gianfranco Biondi, Antonio Torroni, Olga Rickards

**Affiliations:** 1 Department of Biology, University of Rome Tor Vergata, Rome, Italy; 2 Laboratory of Forensic Genetics and Molecular Archaeology, Universitaire Ziekenhuizen, Leuven, Belgium; 3 Center for Archaeological Sciences, Katholieke Universiteit Leuven, Leuven, Belgium; 4 Center for Human Genetics, Katholieke Universiteit Leuven, Leuven, Belgium; 5 Dipartimento di Genetica e Microbiologia, Università di Pavia, Pavia, Italy; 6 Dipartimento di Biologia Cellulare e Ambientale, Università di Perugia, Perugia, Italy; 7 Dipartimento di Scienze Ambientali, Università dell'Aquila, L'Aquila, Italy; Erasmus University Medical Center, Netherlands

## Abstract

The Tuareg of the Fezzan region (Libya) are characterized by an extremely high frequency (61%) of haplogroup H1, a mitochondrial DNA (mtDNA) haplogroup that is common in all Western European populations. To define how and when H1 spread from Europe to North Africa up to the Central Sahara, in Fezzan, we investigated the complete mitochondrial genomes of eleven Libyan Tuareg belonging to H1. Coalescence time estimates suggest an arrival of the European H1 mtDNAs at about 8,000–9,000 years ago, while phylogenetic analyses reveal three novel H1 branches, termed H1v, H1w and H1x, which appear to be specific for North African populations, but whose frequencies can be extremely different even in relatively close Tuareg villages. Overall, these findings support the scenario of an arrival of haplogroup H1 in North Africa from Iberia at the beginning of the Holocene, as a consequence of the improvement in climate conditions after the Younger Dryas cold snap, followed by *in situ* formation of local H1 sub-haplogroups. This process of autochthonous differentiation continues in the Libyan Tuareg who, probably due to isolation and recent founder events, are characterized by village-specific maternal mtDNA lineages.

## Introduction

In the last few years the story of human migrations has been extensively reconstructed thanks to the contribution of archaeology and genetics, particularly the latter through the study of the two uniparentally transmitted genetic systems: mitochondrial DNA (mtDNA) and Y chromosome. The study of maternal genealogies appears to indicate that the peopling of the Eurasian continent by modern humans likely began around 60–70 thousand years ago (kya) through the ‘southern coastal route’ from the Horn of Africa via Arabia and South Asia, up to Australasia [1], [2], [3], [4], [5]. However, alternative exit scenarios, including a more northern route into the Levant and multiple waves of migration (see Kayser 2010 [6] and Majumder 2010 [7] for a review), have recently regained some momentum after the postulated detection of some Neanderthal nuclear DNA variation in the genomes of modern Eurasians [8]. The ‘southern coastal route’ scenario instead implies that, blocked by deserts, humans could not move from the Arabian Gulf area into the Levant earlier than 50 kya, when climate conditions improved [9]. Entrance into the Levant paved the way to the dispersal of modern humans both north-westward into Europe and south-westward into Northern Africa [10], [11]. During the Last Glacial Maximum (LGM), approximately between 26.5 and 19 kya [12] ice sheets largely covered large portions of North America and Europe. In warmer regions of the world, the climate was cooler and drier and deserts spread over large regions, particularly in Northern Africa, Middle East and Central Asia [13], [14]. Accordingly, during the LGM, humans concentrated in refugial areas of southwestern Europe, in the Balkans and Levant, and on the east European plains [9], [15], [16]. The subsequent Bølling warming, around 15 kya, triggered re-expansion processes which led to the resettlement of Central and Northern Europe. Genetic signatures of these expansions are evident in mtDNA genealogies, for instance haplogroups H1, H3 and V contributed to the gradual re-peopling of Europe from the Franco-Cantabrian refuge in the postglacial [17], [18]. Similarly, though to a lesser extent, H5*(xH5a), H20 and H21 may be associated to a postglacial population expansion phase in the Caucasus area [19]. Although restricted to the Mediterranean coast, an expansion took place also from the Italian peninsula northward, as attested to by the haplogroup U5b3 [20].

Evidence of trans-Mediterranean contacts between Northern Africa and Western Europe has been assessed at the level of different genetic markers (e.g. [21], [22], [23], [24]). With regards to the mtDNA, the high incidence of H1 and H3 in Northwest Africa, together with some other West European lineages (i.e. V and U5b), reveals a possible link with the postglacial expansion from the Iberian Peninsula, which not only directed north-eastward into the European continent [17], [18], [25], but also southward, beyond the Strait of Gibraltar, into North Africa [26], [27]. So, besides the ‘autochthonous’ South-Saharan component, the maternal pool of Northern Africa appears to be characterized by at least two other major components: (i) a Levantine contribution (i.e. haplogroups U6 and M1, [11]), associated with the return to Africa around 45 kya, and (ii) a more recent West European input associated with the postglacial expansion.

Within the West-European component in North Africa, H1 is the most represented haplogroup with frequencies ranging from 21% in some Tunisian Berber groups to 1% in Egypt [28]. Recently, an extremely high incidence of H1 (61%) has been reported in a Tuareg population from the Central Sahara, in Libya [29]. Tuareg are a semi-nomadic pastoralist people of Northwest Africa, who speak a Berber language. MtDNA analyses performed on the Libyan Tuareg have highlighted their genetic relatedness with some Berber groups and other North African populations, mainly resulting from the sharing of a common West-Eurasian component. A high degree of homogeneity in the Libyan H1 lineages was observed, suggesting that the high frequency of H1 in the Tuareg may be the result of genetic drift and recent founder events.

To better define the nature and extent of H1 variation in the Tuareg from Libya we have now determined the complete sequence of eleven of their mtDNAs belonging to H1. The comparison of these H1 sequences with those already available from Europe and North Africa provides new clues on how and when H1 spread in Northern Africa up to the Central Sahara.

## Methods

Earlier mtDNA molecular analyses carried out on the two hypervariable segments (HVS-I and HVS-II) and diagnostic markers in the mtDNA coding region in a sample of 129 Libyan Tuareg from two neighboring villages in Fezzan (Al Awaynat and Tahala) allowed the detection of 79 H1 mtDNAs encompassing 61% of the population sample [29]. Appropriate written informed consent to anonymously use their data was obtained from all individuals. The ethics approval for this study was provided by the Ethical committee of the University of Rome Tor Vergata (DM 12 maggio 2006, Delibera n. 243/2007). The genetic diversity of the Libyan H1 mtDNAs appeared to be extremely low, with 91% of the H1 individuals sharing the same HVS-I/II haplotype (i.e. CRS-263). In the present work, a more detailed molecular characterization of the H1 haplotypes was performed. In particular, eight mtDNAs characterized by the CRS-263 haplotype and three mtDNAs harboring the haplotype 16037-16256-263 (for more detail see Ottoni *et al*. 2009 [29]) were chosen and submitted to complete sequencing. The sequencing procedure and the phylogeny construction of the complete mtDNAs were performed as described elsewhere [17], [30].

The *rho* statistic and its standard deviation *sigma* [31] were calculated for both the entire H1 haplogroup and its internal sub-clades. Maximum Likelihood (ML) estimates were also provided by using PAML 3.13 and considering three partitions on the entire mitochondrial molecule: HVS-I (positions 16051 to 16400), HVS-II (positions 68 to 263) and remainder. The age estimates were then converted to years according to the mutation rates of Soares *et al*. [32] and Loogväli *et al*. [33].

Moreover, once the phylogeny of the H1 Tuareg lineages was reconstructed and its internal clades defined, 50 of the 64 remaining Tuareg H1 mtDNAs harboring the CRS-263 haplotype were surveyed for the diagnostic mutations of the novel branches, thus allowing their sub-classification. The remaining 14 mtDNAs were not screened for sub-clade markers due to lack of DNA.

In detail, PCR amplification and sequencing of four fragments was carried out in order to investigate the status at nucleotide positions (nps) 4313 (L4180-AACTTCCTACCACTCACC, H4621-TGGCAGCTTCTGTGGAAC), 9148 (L9003-CCTAACCGCTAACATTAC, H9280-CTAGGCCGGAGGTCATTA), 14560 (L14398-AACACTCACCAAGACCTCAACC, H14832-AGTGAGCCGAAGTTTCATCATG) and 8966 (L8908-TTCTTACCACAAGGCACACC, H9014-TAGGTGGCCTGCAGTAATGT), which respectively mark H1v1, H1v1a, H1v1b and H1w.

The geographic representation of H1 haplogroup frequencies was obtained using Surfer 6 (http://www.goldensoftware.com) with the Kriging procedure. Frequency estimates at each grid node were inferred by considering the entire data set. The extent of H1 variation in North Africa was evaluated from available HVS-I data (nps 16024–16365) with the software Arlequin version 3.1 [34].

## Results

**Figure 1:**
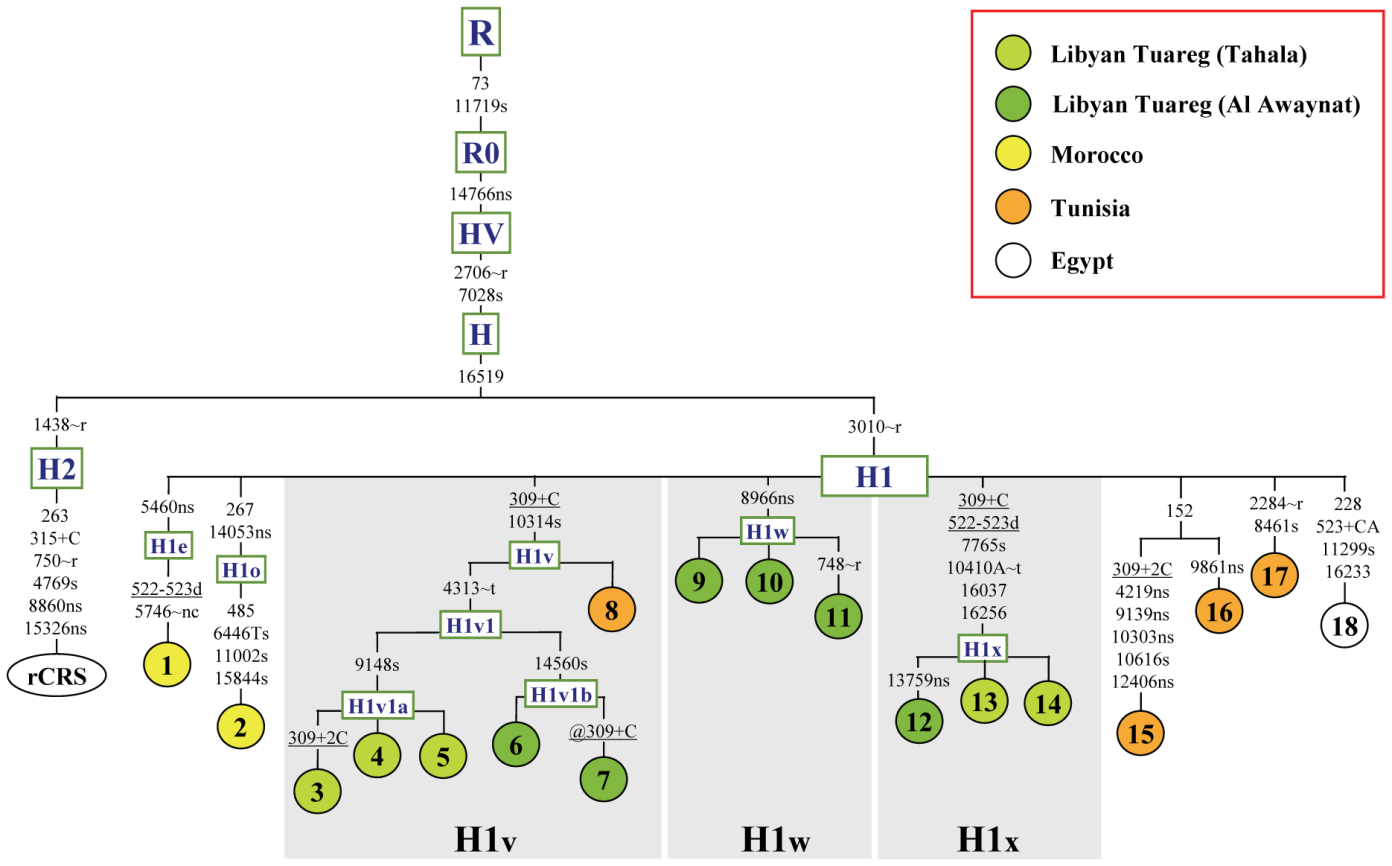
Most parsimonious tree of complete H1 mtDNA sequences from North Africa. The tree includes 18 complete mtDNA sequences and illustrates sub-haplogroup affiliations, including the novel sub-haplogroups H1v, H1w and H1x. Eleven sequences are from the Tuareg of Libya and seven were previously published: four Tunisians, two Moroccan Jews, and one Berber from Egypt (Table S1 and the supplementary References S1). The position of the revised Cambridge reference sequence (rCRS) [47] is indicated for reading off sequence motifs. Tuareg mtDNAs were selected through a preliminary sequence analysis of the control region and an RFLP survey in order to include the widest possible range of internal variation of haplogroup H1. The sequencing procedure and phylogeny construction were performed as described elsewhere [17], [30]. Mutations are shown on the branches; they are transitions unless a base is explicitly indicated. The prefix @ designates reversions, while suffixes indicate: transversions (to A, G, C, or T), indels (+, d), gene locus (∼t, tRNA; ∼r, rRNA; ∼nc, non coding region outside of the mtDNA control region), and synonymous or non-synonymous changes (s or ns). Recurrent mutations are underlined. The root of H1o has been defined according to Behar *et al*. [36]. Additional information regarding each mtDNA is available on Table S1 and on the supplementary References S1. MtDNAs # 1 and 2 are from Moroccan Jews [36], # 3–7 and 9–14 are from Libyan Tuareg, # 8 and 15–17 are from Tunisian subjects [35], and # 18 is from a Berber of Egypt [17].

The most parsimonious tree encompassing eleven complete H1 mtDNAs from the Tuareg together with four previously published sequences from Tunisia [35], one Berber from Egypt [17] and two Jewish Moroccans [36] is illustrated in Figure 1. All Tuareg sequences clustered into three clades that had not been previously reported and thus were termed H1v, H1w and H1x. Five sequences grouped into the sub-clade H1v1 defined by the transition at np 4313. One Tunisian sequence (# 8) did not cluster into H1v1 but was closely related, since it harbored the mutation at 10314 that defines the clade H1v (Figure 1). The sub-clade H1v1 splits into two branches defined by the transitions at np 9148 (clade H1v1a) and 14560 (clade H1v1b). Three Tuareg mtDNAs formed the novel clade H1w that is defined by the transition at np 8966, while the last three Tuareg mtDNAs, apart from the HVS-I transitions at 16037 and 16256, were found to harbor mutations at nps 7765 and 10410 in the coding region (clade H1x). The Tuareg complete mtDNAs have been deposited in GenBank, under the accession numbers reported in Table S1.

Divergence values (*rho* statistics and ML estimates) and the age in years of the most recent common ancestor of the main clusters are reported in Table 1, according to the evolutionary rate estimates described in Soares *et al*. [32] and Loogväli *et al*. [33]. The two evolutionary rates provide a coalescence time of about 8–9 kya for the whole H1 haplogroup in North Africa. As expected, the North African-specific clades are characterized by younger ages ranging from about 3.8 to 6.7 kya for H1v, and from 2.1 to 7.9 kya for H1v1. The youngest clades were found to be H1w and H1x, with an age of about 0.8–1.1 kya.

**Table 1:** Age estimates of relevant nodes in the North African H1 phylogeny illustrated in Figure 1.

		All mutations in the mtDNA				Only synonymous substitutions				Maximum Likelihood (on the entire molecule)			
Clade	No. of mtDNAs	ρ^a^	σ^b^	T (y) c	95% range (y) c	ρ^e^	σ^b^	T (y) f	ΔT (y) f	Substitutions per site	SE	T (y) c	95% range (y) c
H1	18	3.00	0.67	7,892	4,388–11,470	1.11	0.44	8,878	3,551	0.00238	0.00014	9,325	6,862–11,823
H1v	6	1.67	1.03	4,343	0^d^ –9,760	0.83	0.60	6,658	4,801	0.00100	0.00017	3,834	1,014–6,706
> H1v1	5	1.00	0.72	2,585	0^d^ –6,311	1.00	0.72	7,990	5,762	0.00054	0.00021	2,076	0^d^ –5,754
H1w	3	0.33	0.33	848	0^d^ –2,525	0	0	0	0	0.00029	0.00011	1,107	0^d^ –3,027
H1x	3	0.33	0.33	848	0^d^ –2,525	0	0	0	0	0.00021	0.00015	792	0^d^ –3,302

a Average number of base substitutions in the whole mtDNA from the root sequence type excluding 16519 and the 16182C, 16183C and 16194C [32].

b Standard error calculated from an estimate of the genealogy [31].

c Using the corrected molecular clock proposed by Soares et al. [32].

d Negative lower values were ignored.

e Average number of synonymous substitutions in the whole mtDNA from the root sequence type.

f According to the recalibrated synonymous rate of Loogväli et al. [33].

The survey of diagnostic markers 4313, 8966, 9148 and 14560 in 50 individuals characterized by the CRS-263 haplotype showed that all of these clustered within the two novel sub-clades H1v1 and H1w identified by initial sequencing of the eight entire mtDNAs characterized by the CRS-263 control-region motif. Overall the 64 Libyan Tuareg mtDNAs belonging to H1 (Table S2) were mostly distributed between the clades H1v1 (38%) and H1w (53%), with a minor component (9%) belonging instead to clade H1x. Within H1v1, half of the Libyan Tuareg (i.e. 12 individuals, equal to 50%) were characterized by the transition at np 9148 (sub-clade H1v1a) and half by the transition at np 14560 (sub-clade H1v1b). It is worth noting the extensive village-specificity of the sub-clades. Indeed H1v1b and H1w harbored frequencies of 22% and 63% in Al Awaynat, but were not found at all in Tahala, and 80% of the mtDNAs from the village of Tahala were members of H1v1a in contrast to the only four out of 54 (7%) from the village of Al Awaynat. Similar to H1v1a, haplogroup H1x was also shared between the two groups with two instances in Tahala and four in Al Awaynat.

**Figure 2:**
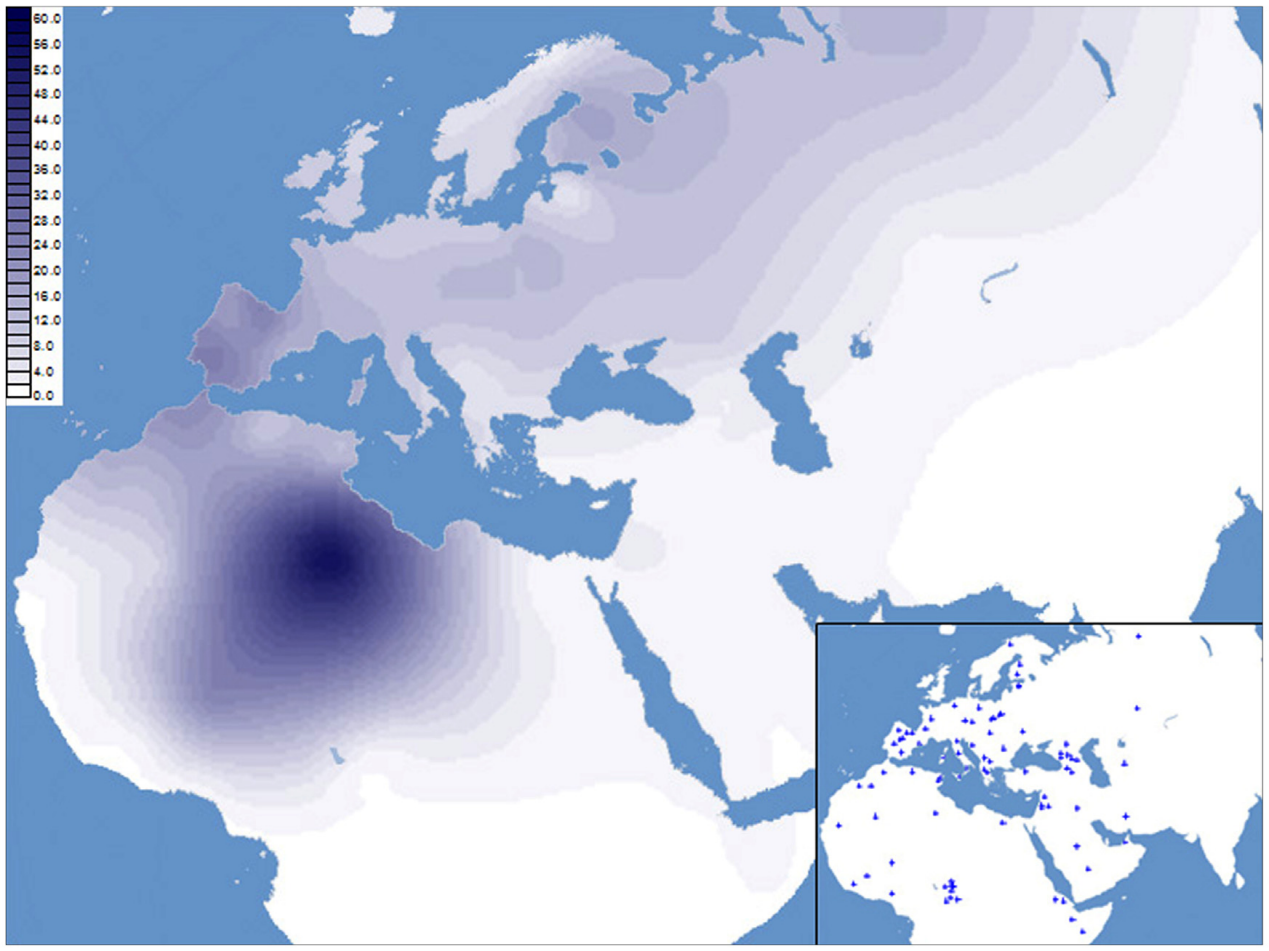
Spatial frequency distribution (%) of haplogroup H1 in western Eurasia and North Africa. The inset illustrates the geographic location of populations surveyed. Populations and corresponding frequency values are listed in Table S3 and in the supplementary References S1.

**Figure 3:**
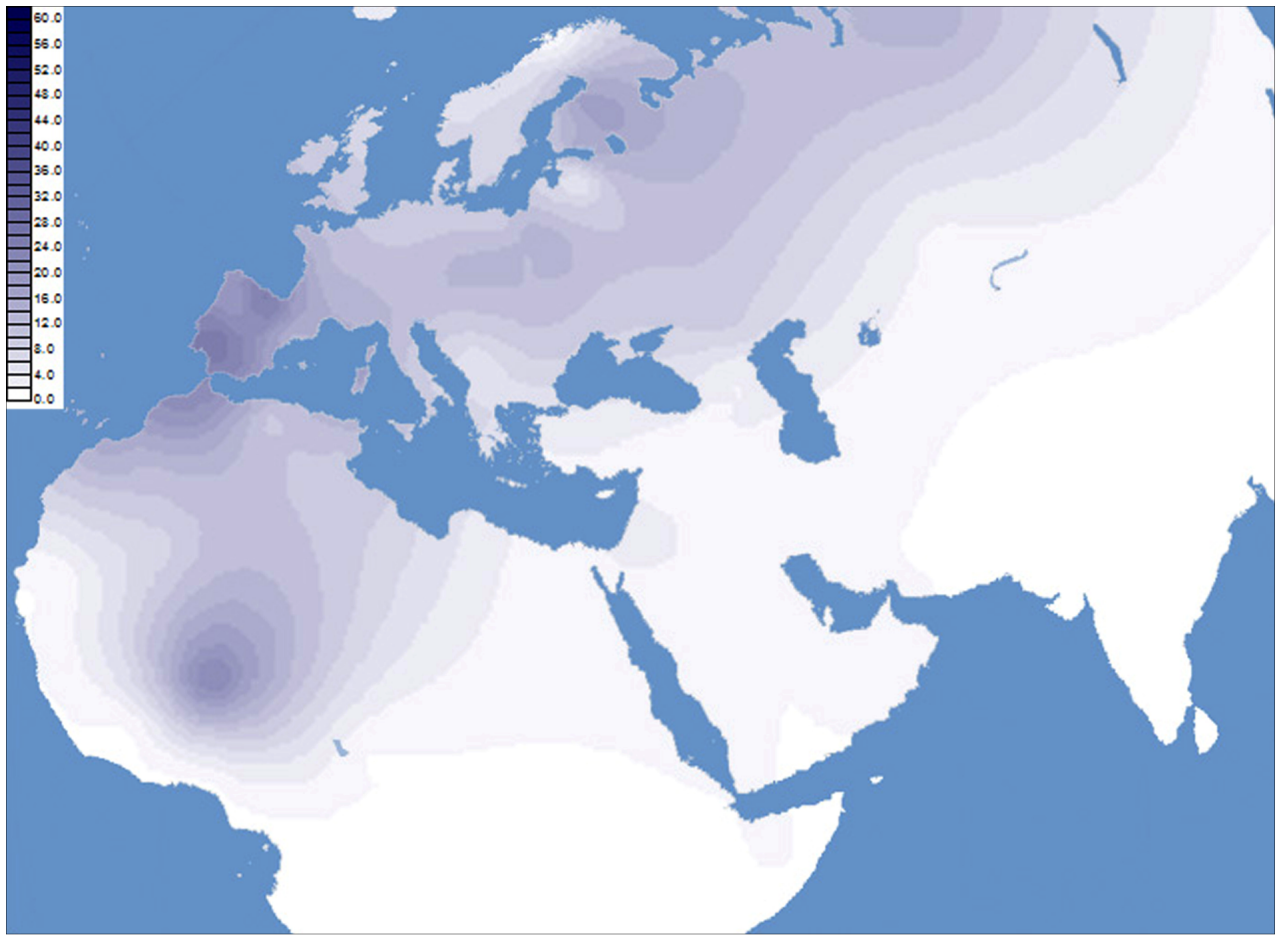
Spatial frequency distribution (%) of haplogroup H1 in western Eurasia and North Africa after excluding the Libyan Tuareg samples.

An up-to-date map of the H1 spatial distribution in Africa and West Eurasia is illustrated in Figure 2. Frequency data and other details of the populations from the literature included in this survey are reported in Table S3 and in the supplementary References S1. There is an evident frequency peak in the Central Sahara associated with the Libyan Tuareg, who show the highest frequency value (61%) among all the populations considered in the analysis. Since the high frequency of H1 in the Libyan Tuareg is most likely the result of random genetic drift and founder events, we also investigated the H1 distribution removing the Libyan Tuareg sample and thus leaving only previously reported data (Figure 3). As expected, frequency peaks in the European continent were observed in the Iberian Peninsula, whereas in Northern Africa the rather high frequency values in Morocco and Tunisia became apparent. More southward, among the Tuareg from the Sahel region [37], a frequency peak is also observed. To further evaluate the extent of H1 variation in the Tuareg from Libya relative to that of Moroccans, Tunisians and Sahelian Tuareg samples, HVS-I data from the four groups were employed to calculate the diversity indices reported in Table 2. The sharp homogeneity of H1 in the Libyan Tuareg, who show extremely low values of haplotype diversity (0.165), is straightforward. Moroccans, Tunisians and the Tuareg from Sahel were found to be much more diverse than the Libyan Tuareg, with haplotype diversities of 0.577, 0.633 and 0.595, respectively. Similarly, the values of nucleotide diversity and average number of nucleotide differences observed in Morocco (0.309 and 1.056), Tunisia (0.316 and 1.081) and among the Tuareg from Sahel (0.234 and 0.800) are all much higher than those of the Libyan Tuareg (0.098 and 0.335).

**Table 2:** Diversity of haplogroup H1 in North African populations.

	No. of mtDNAs	No. of haplotypes^a^	H^b^	π^c^	M^d^	Ref.
Libyan Tuareg	79	3	0.165±0.054	0.098±0.101	0.335±0.338	[29]
Tuareg (West Sahel)	21	5	0.595±0.101	0.234±0.196	0.800±0.601	[37]
Morocco	80	22	0.577±0.067	0.309±0.229	1.056±0.708	[28], [48]
Tunisia	56	19	0.633±0.076	0.316±0.234	1.081±0.723	[27], [48]

a HVS-I haplotypes (from np 16024 to np 16365).

b Haplotype diversity.

c Nucleotide diversity %.

d Average number of nucleotide differences.

## Discussion

The H1 phylogeny based on complete North African sequences reveals a degree of branch diversification that is almost undetectable when using only control region data. Moreover, when compared to the H1 phylogeny built using complete sequences from Europe [17], [19], [38], [39], it appears that the novel branches H1v, H1w and H1x identified during the course of this study are all African-specific. This finding suggests that these H1 sub-clades most likely arose in North Africa after the arrival of the H1 European founder sequence, corresponding to the H1 node in Figure 1. The issue of the North African specificity of H1v, H1w and H1x needs to be corroborated by additional survey of H1 variation in Western Europe, especially in Iberia, but for the moment none of the European complete mtDNA sequences belonging to H1 were found to belong to these clades. This scenario is further supported by the overall age of haplogroup H1 in North Africa. Using the evolution rates recently proposed by Soares *et al*. [32] and Loogväli *et al*. [33], haplogroup H1 shows a coalescence time of approximately 8–9 ky (Table 1), in agreement with the hypothesis of an early arrival and radiation of H1 in the African continent in the first half of the Holocene, as a consequence of the postglacial expansion from the Iberian Peninsula. An arrival from Iberia explains the extent of H1 variation observed in North African populations (Table 2). Indeed, Moroccans and Tunisians, the populations geographically closest to Europe, harbor the highest diversity values for all considered indices. Thus, the coastal areas of northwestern Africa, after the arrival of the Iberian founder H1 mtDNAs, probably acted as centers for the subsequent diffusion of H1 in the internal regions of North Africa. The rather high frequency of H1 in the Tuareg from Sahel (23.3%), in association with intermediate diversity values, is in agreement with the proposal that drift played a major role in shaping the genetic structure of inland populations after they were entrapped in the Sahel belt by the desertification of the Sahara [37]. As for the Libyan Tuareg, the extremely low values of the diversity indices confirm that the outstanding high frequency of H1 in this population is the result of even more recent founder events.

The H1 phylogeny shows a link between the Tuareg of Libya and one Tunisian at the level of clade H1v (Figure 1). Therefore, the H1v coalescence time of about 4–7 ky (Table 1) might correspond to an ancestral split within a nomadic population of Northwest Africa, which led also to the formation of a derived Central Saharan population. The H1v1 sub-clade most likely arose in this population of Central Sahara, which, in turn, contributed extensively to the mtDNA pool of the modern villages of Tahala and Al Awaynat. However, the distribution of the H1v1 lineages in the two Tuareg villages, with H1v1b found only in Al Awaynat and 80% of H1v1a in Tahala and only 7% in Al Awaynat, indicates a rather sharp distinction and a village-specificity of the modern mtDNA gene pool. This is probably the result of peculiar long lasting cultural practices in the Tuareg, who define the affiliation to tribes by matrilineal descent [40], and points to a high degree of isolation between the two villages, at least at the maternal level, regardless of their geographic proximity.

The migratory dynamics which took place over the last 2 ky in the hyperarid Central Sahara, and which possibly led small Tuareg groups with different maternal ancestries to mix and separate from one another [29], [41], [42], could explain the presence of the other two clades, H1w and H1x (respectively 53% and 9% of the total H1 mtDNAs analyzed), in the Libyan Tuareg sample. It should be noted that historically this phase coincides with the decline of the Garamantes, whom the Tuareg consider as their ancestors according to oral traditions [43], [44], [45]. These people inhabited the Fezzan between 2.7 and 1.8 kya and established state-entities based on sedentary settlements and the trans-Saharan caravan trade system. It is plausible that the dismantling of the Garamantian society led small groups to separate as distinct tribes, or alternatively to blend into larger groups.

The French colonization, in the early 20^th^ century, might have also determined the admixture of different Tuareg groups. Indeed, when the Tuareg were confined to restricted areas, there was a decline in their socio-political system and a forced mixture of previously separated tribal groups [46]. Nevertheless, the hypothesis that the three H1 clusters detected here in the Tuareg were all present in the same founder group cannot be totally ruled out, particularly since we are dealing with a small and isolated human group in which genetic drift might have significantly affected the make-up of the mitochondrial pool.

Overall, the results of this study support the hypothesis that most of the West Eurasian maternal contribution detectable in Northwest African populations is likely linked to prehistoric (i.e. the post-glacial expansion from the Iberian Peninsula) rather than more recent historic events [26], [27], [37]. Furthermore, the data presented confirm that the analysis of complete mtDNA sequences represents a valuable tool to reveal not only the spatial patterns beneath large-scale colonization events, but also those of smaller-scale dispersals which may have contributed to the origin of modern populations. In this regard, additional efforts in the full mtDNA analyses of nomad Northern African populations might resolve the debate concerning their origin and their mutual relationship.

## Supporting Information

Table S1 List of H1 complete sequences included in Figure 1.
(0.04 MB DOC)

Table S2 Assignment of the 64 H1 mtDNAs detected in the Libyan Tuareg to sub-haplogroups H1v, H1w and H1x. Diagnostic sites in the coding region are also reported. Control region data are from Ottoni et al. [5].
(0.09 MB DOC)

Table S3 Frequencies of haplogroup H1 in the population samples included in Figures 2 and 3.
(0.09 MB DOC)

References S1 (0.03 MB DOC)
