# Supplementary material for: Mitochondrial Haplogroup H1 in North Africa: An Early Holocene Arrival from Iberia
Source: PLoS One. 2010 Oct 21;5(10):e13378. doi: 10.1371/journal.pone.0013378 (PMC2958834; doi:10.1371/journal.pone.0013378)
Supplement: Table S1 — List of H1 complete sequences included in Figure 1. (0.04 MB DOC) [file pone.0013378.s001.doc]

**Table S1.** List of H1 complete sequences included in Figure 1.

| **Sequence ID ^a^** | **Haplogroup** | **Geographic/Ethnic Origin** | **GenBank ID** | **References** |
| --- | --- | --- | --- | --- |
| 1 | H1e | Morocco (Jew) | EF556181 | [1] |
| 2 | H1o | Morocco (Jew) | EF556177 | [1] |
| 3 | H1v1a | Libya – Tahala (Tuareg) | HM171270 | [2] |
| 4 | H1v1a | Libya – Tahala (Tuareg) | HM171271 | [2] |
| 5 | H1v1a | Libya – Tahala (Tuareg) | HM171272 | [2] |
| 6 | H1v1b | Libya – Al Awaynat (Tuareg) | HM171273 | [2] |
| 7 | H1v1b | Libya – Al Awaynat (Tuareg) | HM171274 | [2] |
| 8 | H1v | Tunisia | FJ460532 | [3] |
| 9 | H1w | Libya – Al Awaynat (Tuareg) | HM171275 | [2] |
| 10 | H1w | Libya – Al Awaynat (Tuareg) | HM171276 | [2] |
| 11 | H1w | Libya – Al Awaynat (Tuareg) | HM171277 | [2] |
| 12 | H1x | Libya – Al Awaynat (Tuareg) | HM171278 | [2] |
| 13 | H1x | Libya – Tahala (Tuareg) | HM171279 | [2] |
| 14 | H1x | Libya – Tahala (Tuareg) | HM171280 | [2] |
| 15 | H1 | Tunisia | FJ460548 | [3] |
| 16 | H1 | Tunisia | FJ460534 | [3] |
| 17 | H1 | Tunisia | FJ460544 | [3] |
| 18 | H1 | Egypt – Siwa (Berber) | AY738973 | [4] |

^a^ ID numbers correspond to the numbers in Figure 1.
