# Supplementary material for: Mitochondrial Haplogroup H1 in North Africa: An Early Holocene Arrival from Iberia
Source: PLoS One. 2010 Oct 21;5(10):e13378. doi: 10.1371/journal.pone.0013378 (PMC2958834; doi:10.1371/journal.pone.0013378)
Supplement: Table S2 — Assignment of the 64 H1 mtDNAs detected in the Libyan Tuareg to sub-haplogroups H1v, H1w and H1x. Diagnostic sites in the coding region are also reported. Control region data are from Ottoni et al. [5]. (0.09 MB DOC) [file pone.0013378.s002.doc]

Table S2. Assignment of the 64 H1 mtDNAs detected in the Libyan Tuareg to sub-haplogroups H1v, H1w and H1x. Diagnostic sites in the coding region are also reported. Control region data are from Ottoni *et al*. [5].

| **Sample ID** | **Control Region**  **(HVS-I/HVS-II)** | **4313** | **9148** | **14560** | **8966** | **Haplogroup** |
| --- | --- | --- | --- | --- | --- | --- |
|  |  | T ^a^ | T ^a^ | G^a^ | T ^a^ |  |
| Aw 3 | CRS/263 | C | C |  |  | H1v1a |
| Aw 33 | CRS/263 | C | C |  |  | H1v1a |
| Aw 94 | CRS/263 | C | C |  |  | H1v1a |
| Aw 108 | CRS/263 | C | C |  |  | H1v1a |
| Tah 2 | CRS/263 | C | C |  |  | H1v1a |
| Tah 4 ^b^ | CRS/263 | C | C |  |  | H1v1a |
| Tah 10 ^b^ | CRS/263 | C | C |  |  | H1v1a |
| Tah 11 | CRS/263 | C | C |  |  | H1v1a |
| Tah 15 | CRS/263 | C | C |  |  | H1v1a |
| Tah 16 ^b^ | CRS/263 | C | C |  |  | H1v1a |
| Tah 17 | CRS/263 | C | C |  |  | H1v1a |
| Tah 18 | CRS/263 | C | C |  |  | H1v1a |
| Aw 11 | CRS/263 | C | T | A |  | H1v1b |
| Aw 20 | CRS/263 | C | T | A |  | H1v1b |
| Aw 27 | CRS/263 | C | T | A |  | H1v1b |
| Aw 41 | CRS/263 | C | T | A |  | H1v1b |
| Aw 59 | CRS/263 | C | T | A |  | H1v1b |
| Aw 66 | CRS/263 | C | T | A |  | H1v1b |
| Aw 70 | CRS/263 | C | T | A |  | H1v1b |
| Aw 81 ^b^ | CRS/263 | C | T | A |  | H1v1b |
| Aw 83 | CRS/263 | C | T | A |  | H1v1b |
| Aw 86 | CRS/263 | C | T | A |  | H1v1b |
| Aw 89 ^b^ | CRS/263 | C | T | A |  | H1v1b |
| Aw 93 | CRS/263 | C | T | A |  | H1v1b |
| Aw 5 ^b^ | CRS/263 | T |  |  | C | H1w |
| Aw 13 | CRS/263 | T |  |  | C | H1w |
| Aw 31 | CRS/263 | T |  |  | C | H1w |
| Aw 35 | CRS/263 | T |  |  | C | H1w |
| Aw 36 | CRS/263 | T |  |  | C | H1w |
| Aw 39 | CRS/263 | T |  |  | C | H1w |
| Aw 40 | CRS/263 | T |  |  | C | H1w |
| Aw 46 | CRS/263 | T |  |  | C | H1w |
| Aw 47 | CRS/263 | T |  |  | C | H1w |
| Aw 48 | CRS/263 | T |  |  | C | H1w |
| Aw 49 ^b^ | CRS/263 | T |  |  | C | H1w |
| Aw 52 | CRS/263 | T |  |  | C | H1w |
| Aw 54 | CRS/263 | T |  |  | C | H1w |
| Aw 55 ^b^ | CRS/263 | T |  |  | C | H1w |
| Aw 61 | CRS/263 | T |  |  | C | H1w |
| Aw 63 | CRS/263 | T |  |  | C | H1w |
| Aw 65 | CRS/263 | T |  |  | C | H1w |
| Aw 71 | CRS/263 | T |  |  | C | H1w |
| Aw 76 | CRS/263 | T |  |  | C | H1w |
| Aw 82 | CRS/263 | T |  |  | C | H1w |
| Aw 84 | CRS/263 | T |  |  | C | H1w |
| Aw 87 | CRS/263 | T |  |  | C | H1w |
| Aw 88 | CRS/263 | T |  |  | C | H1w |
| Aw 90 | CRS/263 | T |  |  | C | H1w |
| Aw 95 | CRS/263 | T |  |  | C | H1w |
| Aw 97 | CRS/263 | T |  |  | C | H1w |
| Aw 98 | CRS/263 | T |  |  | C | H1w |
| Aw 100 | CRS/263 | T |  |  | C | H1w |
| Aw 103 | CRS/263 | T |  |  | C | H1w |
| Aw 104 | CRS/263 | T |  |  | C | H1w |
| Aw 105 | CRS/263 | T |  |  | C | H1w |
| Aw 106 | CRS/263 | T |  |  | C | H1w |
| Aw 109 | CRS/263 | T |  |  | C | H1w |
| Aw 110 | CRS/263 | T |  |  | C | H1w |
| Aw 32 | 16037-16256/263 |  |  |  |  | H1x |
| Aw 53 ^b^ | 16037-16256/263 |  |  |  |  | H1x |
| Aw 68 | 16037-16256/263 |  |  |  |  | H1x |
| Aw 78 | 16037-16256/263 |  |  |  |  | H1x |
| Tah 3 ^b^ | 16037-16256/263 |  |  |  |  | H1x |
| Tah 13 ^b^ | 16037-16256/263 |  |  |  |  | H1x |

^a^ CRS.

^b^ The entire mtDNA sequence was determined.
