# Supplementary material for: Mitochondrial Haplogroup H1 in North Africa: An Early Holocene Arrival from Iberia
Source: PLoS One. 2010 Oct 21;5(10):e13378. doi: 10.1371/journal.pone.0013378 (PMC2958834; doi:10.1371/journal.pone.0013378)
Supplement: Table S3 — Frequencies of haplogroup H1 in the population samples included in Figures 2 and 3. (0.09 MB DOC) [file pone.0013378.s003.doc]

**Table S3.** Frequencies of haplogroup H1 in the population samples included in Figures 2 and 3.

| **Region or Population** | **H1 Frequency (%)** | | **No. of subjects** | **References** |
| --- | --- | --- | --- | --- |
| Africa: | |  |  |  |
| Libyan Tuareg | | 61.0 | 129 | [5] |
| Tuareg (West Sahel) | | 23.3 | 90 | [6] |
| Berbers (Morocco) | | 20.2 | 217 | [7] |
| Morocco | | 12.2 | 180 | [8] |
| Berbers (Tunisia) | | 13.4 | 276 | [9] |
| Tunisia | | 10.6 | 269 | [4,8] |
| Algeria | | 9.8 | 80 | [4] |
| Berbers (Egypt) | | 1.1 | 184 | [4,7,10] |
| Western Sahara | | 14.8 | 128 | [8] |
| Mauritania | | 6.9 | 102 | [8] |
| Senegal | | 0 | 100 | [4] |
| Fulani (Chad-Cameroon) | | 0 | 186 | [11] |
| Cameroon | | 0 | 142 | [12] |
| Chad | | 0 | 77 | [11] |
| Buduma (Niger) | | 0 | 30 | [11] |
| Nigeria | | 0 | 69 | [11] |
| Ethiopia | | 0 | 82 | [13] |
| Amhara (Ethiopia) | | 0 | 90 | [14] |
| Oromo (Ethiopia) | | 0 | 117 | [13,14] |
| Sierra Leone | | 0 | 155 | [15] |
| Guineans (Guiné Bissau) | | 0 | 372 | [16] |
| Mali | | 0 | 83 | [17] |
| Kikuyu (Kenya) | | 0 | 24 | [18] |
| Benin | | 0 | 192 | [14] |
| Asia: | |  |  |  |
| Central Asia | | 0.7 | 445 | [19] |
| Pakistan | | 0 | 100 | [4] |
| Yakutia | | 1.7 | 58 | [4] |
| Caucasus: | |  |  |  |
| Caucasus (north) | | 8.8 | 68 | [4] |
| Caucasus (south) | | 2.3 | 132 | [4] |
| Northwestern Caucasus | | 4.7 | 234 | [20] |
| Armenians | | 2.3 | 175 | [20] |
| Daghestan | | 2.5 | 269 | [20] |
| Georgians | | 1.0 | 193 | [20] |
| Karatchaians-Balkarians | | 4.4 | 203 | [20] |
| Ossetians | | 2.4 | 296 | [20] |
| Europe: | |  |  |  |
| Andalusia | | 24.3 | 103 | [4] |
| Basques (Spain) | | 27.8 | 108 | [4] |
| Catalonia | | 13.9 | 101 | [21] |
| Galicia | | 17.7 | 266 | [22] |
| Pasiegos (Cantabria) | | 23.5 | 51 | [4] |
| Portugal | | 25.5 | 499 | [23] |
| Spain (miscellaneous) | | 18.9 | 132 | [4] |
| Italy (north) | | 11.5 | 322 | [4] |
| Italy (center) | | 6.3 | 208 | [4] |
| Italy (south) | | 8.7 | 206 | [4] |
| Sardinia | | 17.9 | 106 | [4] |
| Sicily | | 10.0 | 90 | [4] |
| Finland | | 18.0 | 78 | [19] |
| Volga-Ural Finnic speakers | | 13.6 | 125 | [19] |
| Basques (France) | | 17.5 | 40 | [4] |
| Béarnaise | | 14.8 | 27 | [4] |
| France | | 12.3 | 106 | [19] |
| Estonia | | 16.7 | 114 | [19] |
| Saami | | 0 | 57 | [4] |
| Lithuania | | 1.7 | 180 | [24] |
| Hungary | | 11.3 | 303 | [4,25] |
| Czech Republic | | 10.8 | 102 | [4] |
| Ukraine | | 9.9 | 191 | [4,19] |
| Poland | | 9.3 | 86 | [4] |
| Russia | | 13.5 | 312 | [19] |
| Austria | | 10.6 | 2487 | [25] |
| Germany | | 6.0 | 100 | [25] |
| Romania | | 9.4 | 360 | [25] |
| Netherlands | | 8.8 | 34 | [4] |
| Greece (Aegean islands) | | 1.6 | 247 | [4] |
| Greece (mainland) | | 6.3 | 79 | [4] |
| Macedonia | | 7.1 | 252 | [4,25] |
| Albania | | 2.9 | 105 | [4] |
| Balkans | | 5.4 | 111 | [19] |
| Croatia | | 8.3 | 84 | [4] |
| Slovaks | | 7.6 | 119 | [19] |
| Slovak (East) | | 16.8 | 137 | [26] |
| Slovak (West) | | 14.2 | 70 | [26] |
| Middle East: | |  |  |  |
| Arabian Peninsula | | 0 | 94 | [4] |
| Arabian Peninsula (incl. Yemen, Oman) | | 0.8 | 493 | [20] |
| Turks | | 3.3 | 360 | [20] |
| Druze | | 3.4 | 58 | [4] |
| Dubai (United Arab Emirates) | | 0.4 | 249 | [27] |
| Iraq | | 1.9 | 206 | [4] |
| Jordanians | | 1.7 | 173 | [20] |
| Lebanese | | 4.2 | 167 | [20] |
| Syrians | | 0 | 159 | [20] |
