# Supplementary material for: Mitochondrial Haplogroup H1 in North Africa: An Early Holocene Arrival from Iberia
Source: PLoS One. 2010 Oct 21;5(10):e13378. doi: 10.1371/journal.pone.0013378 (PMC2958834; doi:10.1371/journal.pone.0013378)
Supplement: References S1 — (0.03 MB DOC) [file pone.0013378.s004.doc]

**Supporting Information References**

1. Behar DM, Metspalu E, Kivisild T, Rosset S, Tzur S, et al. (2008) Counting the founders: the matrilineal genetic ancestry of the Jewish Diaspora. PLoS One 3: e2062.

2. This study.

3. Costa MD, Cherni L, Fernandes V, Freitas F, Ammar El Gaaied AB, et al. (2009) Data from complete mtDNA sequencing of Tunisian centenarians: testing haplogroup association and the "golden mean" to longevity. Mech Ageing Dev 130: 222-226.

4. Achilli A, Rengo C, Magri C, Battaglia V, Olivieri A, et al. (2004) The molecular dissection of mtDNA haplogroup H confirms that the Franco-Cantabrian glacial refuge was a major source for the European gene pool. Am J Hum Genet 75: 910-918.

5. Ottoni C, Martinez-Labarga C, Loogväli EL, Pennarun E, Achilli A, et al. (2009) First genetic insight into Libyan Tuaregs: a maternal perspective. Ann Hum Genet 73: 438-448.

6. Pereira L, Cerny V, Cerezo M, Silva NM, Hajek M, et al. (2010) Linking the sub-Saharan and West Eurasian gene pools: maternal and paternal heritage of the Tuareg nomads from the African Sahel. Eur J Hum Genet. Eur J Hum Genet 18: 915-923

7. Coudray C, Olivieri A, Achilli A, Pala M, Melhaoui M, et al. (2009) The complex and diversified mitochondrial gene pool of Berber populations. Ann Hum Genet 73: 196-214.

8. Ennafaa H, Cabrera VM, Abu-Amero KK, Gonzalez AM, Amor MB, et al. (2009) Mitochondrial DNA haplogroup H structure in North Africa. BMC Genet 10: 8.

9. Cherni L, Fernandes V, Pereira JB, Costa MD, Goios A, et al. (2009) Post-last glacial maximum expansion from Iberia to North Africa revealed by fine characterization of mtDNA H haplogroup in Tunisia. Am J Phys Anthropol 139: 253-260.

10. Kujanova M, Pereira L, Fernandes V, Pereira JB, Cerny V (2009) Near Eastern Neolithic genetic input in a small oasis of the Egyptian Western Desert. Am J Phys Anthropol 140: 336-346.

11. Cerny V, Salas A, Hajek M, Zaloudkova M, Brdlcka R (2007) A bidirectional corridor in the Sahel-Sudan belt and the distinctive features of the Chad basin populations: a history revealed by the mitochondrial DNA genome. Ann Hum Genet 71: 433-452.

12. Cerny V, Hajek M, Cmejla R, Bruzek J, Brdicka R (2004) mtDNA sequences of Chadic-speaking populations from northern Cameroon suggest their affinities with eastern Africa. Ann Hum Biol 31: 554-569.

13. Kivisild T, Reidla M, Metspalu E, Rosa A, Brehm A, et al. (2004) Ethiopian mitochondrial DNA heritage: tracking gene flow across and around the gate of tears. Am J Hum Genet 75: 752-770.

14. Rickards O, unpulished

15. Jackson BA, Wilson JL, Kirbah S, Sidney SS, Rosenberger J, et al. (2005) Mitochondrial DNA genetic diversity among four ethnic groups in Sierra Leone. Am J Phys Anthropol 128: 156-163.

16. Rosa A, Brehm A, Kivisild T, Metspalu E, Villems R (2004) MtDNA profile of West Africa Guineans: towards a better understanding of the Senegambia region. Ann Hum Genet 68: 340-352.

17. Gonzalez AM, Cabrera VM, Larruga JM, Tounkara A, Noumsi G, et al. (2006) Mitochondrial DNA variation in Mauritania and Mali and their genetic relationship to other Western Africa populations. Ann Hum Genet 70: 631-657.

18. Watson E, Bauer K, Aman R, Weiss G, von Haeseler A, et al. (1996) mtDNA sequence diversity in Africa. Am J Hum Genet 59: 437-444.

19. Loogväli EL, Roostalu U, Malyarchuk BA, Derenko MV, Kivisild T, et al. (2004) Disuniting uniformity: A pied cladistic canvas of mtDNA Haplogroup H in Eurasia. Mol Biol Evol 21: 2012-2021.

20. Roostalu U, Kutuev I, Loogväli EL, Metspalu E, Tambets K, et al. (2007) Origin and expansion of haplogroup H, the dominant human mitochondrial DNA lineage in West Eurasia: the Near Eastern and Caucasian perspective. Mol Biol Evol 24: 436-448.

21. Alvarez-Iglesias V, Mosquera-Miguel A, Cerezo M, Quintans B, Zarrabeitia MT, et al. (2009) New population and phylogenetic features of the internal variation within mitochondrial DNA macro-haplogroup R0. PLoS One 4: e5112.

22. Quintans B, Alvarez-Iglesias V, Salas A, Phillips C, Lareu MV, et al. (2004) Typing of mitochondrial DNA coding region SNPs of forensic and anthropological interest using SNaPshot minisequencing. Forensic Sci Int 140: 251-257.

23. Rosa A, Fonseca B, Krug T, Manso H, Gouveia L, et al. (2008) Mitochondrial haplogroup H1 is protective for ischemic stroke in Portuguese patients. BMC Med Genet 9: 57

24. Kasperaviciute D, Kucinskas V, Stoneking M (2004) Y chromosome and mitochondrial DNA variation in Lithuanians. Ann Hum Genet 68: 438-452.

25. Brandstätter A, Zimmermann B, Wagner J, Göbel T, Röck A, et al. (2008) Timing and deciphering mitochondrial DNA macro-haplogroup R0 variability in Central Europe and Middle East. BMC Evol Biol 8: 191.

26. Malyarchuk BA, Perkova MA, Derenko MV, Vanecek T, Lazur J, et al. (2008) Mitochondrial DNA variability in Slovaks, with application to the Roma origin. Ann Hum Genet 72: 228-240.

27. Alshamali F, Brandstatter A, Zimmermann B, Parson W (2008) Mitochondrial DNA control region variation in Dubai, United Arab Emirates. Forensic Sci Int Genet 2: e9-10.
